# Is Regional Bone Mineral Density the Differentiating Factor Between Femoral Neck and Femoral Trochanteric Fractures?

**DOI:** 10.7759/cureus.53003

**Published:** 2024-01-26

**Authors:** Christos Vlachos, Margarita-Michaela Ampadiotaki, Eftychios Papagrigorakis, Athanasios Galanis, Christos Patilas, Evangelos Sakellariou, Georgios Rodis, Elias Vasiliadis, Vasileios A Kontogeorgakos, Spiros Pneumaticos, John Vlamis

**Affiliations:** 1 3rd Orthopedic Department, National and Kapodistrian University of Athens, KAT General Hospital, Athens, GRC; 2 2nd Orthopedic Department, KAT Attica General Hospital, Athens, GRC; 3 Radiology Department, KAT General Hospital, Athens, GRC; 4 3rd Orthopedic Department, National and Kapodistrian University of Athens, KAT Trauma Hospital, Athens, GRC; 5 Orthopedics Department, Attikon General University Hospital, Athens, GRC

**Keywords:** t-score, femoral trochanteric fractures, femoral neck fractures, bone mass density, proximal femur fracture

## Abstract

Background

Osteoporosis is globally recognized as a prevalent bone disease, and proximal femoral fractures constitute a serious complication associated with it. In recent years, the frequency of hip fractures has increased rapidly, with ramifications that extend into the social and economic aspects of both patients’ lives and healthcare systems. The primary goal of this study is to discover whether bone mineral density (BMD) in specific regions of the hip could be related to femoral neck or trochanteric fractures.

Methodology

This prospective cohort study employed dual-energy X-ray absorptiometry (DEXA) measurements on 70 individuals with proximal femoral fractures. The participants sought treatment at the emergency department of our unit for hip fractures and adhered to our predefined eligibility criteria. These criteria primarily included (i) age exceeding 60 years and (ii) a diagnosis of either femoral neck or trochanteric fracture attributed to (iii) a low-energy lateral fall and (iv) a previously established state of complete ambulation before the occurrence of the fracture. In this context, we recorded the BMD of the hip, as well as the BMD values of the upper and lower halves of the neck, trochanteric region, and diaphysis. For the comparison of the categorical variables, Pearson’s χ^2^ criterion was used, whereas Student’s t-test was applied for the comparison of means of quantitative variables across fracture types.

Results

No statistical differences were identified when comparing regional BMDs and T-scores with the fracture type. This conclusion was also reconfirmed concerning age, gender, and Tonnis classification. Only a moderate correlation was observed, demonstrating lower values of regional BMDs in women compared to men.

Conclusions

The inability of our study to establish a direct correlation between BMD measurements across diverse areas of the proximal femur underlines the imperative need for subsequent investigations. These studies should not only integrate more precise techniques for measuring and mapping the BMD of different hip regions but should also encompass a comprehensive examination that would consider both intrinsic and extrinsic characteristics of the proximal femur.

## Introduction

Osteoporosis, both primary and secondary, is widely recognized as one of the most prevalent bone diseases globally [1]. The elevated occurrence of the subsequent osteoporotic fractures, exacerbated by muscle atrophy and neurological conditions, has, in turn, identified them as a leading cause of mortality among the elderly population [2]. The typical areas of these fractures, from higher to lower incidence, encompass the vertebrae, the hip, and the distal radius [2-4]. More precisely, the projected lifetime risk of experiencing an osteoporotic fracture in the United States after the fifth decade of life is approximately 40% for females and 13% for males [5]. In the context of our study, fragility fractures of the proximal femur contribute to an overall incidence of mortality of up to 69.38%. This is further delineated as 33% within the first 12 months and an additional 2% for each subsequent year [6].

The frequency of osteoporotic hip fractures is anticipated to escalate due to the recent advancements in medicine and pharmacology, both of which have contributed to an increased average life expectancy [7,8]. In 1990, the reported total number of hip fractures was 1.7 million, with an estimated increase of up to 6.3 million by 2050 [9]. Moreover, the reverberations of hip fractures extend beyond the diminished well-being of the affected individuals and their families, exerting an unsustainable financial strain on healthcare systems worldwide [10,11]. Of note, the immediate treatment costs per patient account for approximately 7,000$ and increase to up to 21,000$ during the first year of rehabilitation. Notably, only half of these patients are expected to regain their preoperative levels of mobility and independence [9].

Hip fractures are anatomically classified as either femoral neck (FNF) or femoral trochanteric (FTF). It is worth mentioning that 62% of femoral neck and 72% of trochanteric fractures are preceded by a contralateral fracture of the identical type [12-14]. This observation emphasizes the possibility that specific characteristics are associated with each type of fracture. Several studies have attempted to investigate the relationship between proximal femur geometry and hip fracture type, with conflicting results. As a result, recent studies are increasingly focusing on the intrinsic features of the proximal femur, such as bone microarchitecture and bone mineral density (BMD).

Notably, the human skeleton is in a constant state of remodeling, a process planned to repair and replace existing bone tissue. Yet, as a person ages, this regenerative capacity gradually declines, leading to a significant loss of bone mass and subsequent changes in the bone microarchitecture. When BMD falls below a critical threshold, it becomes quantitatively very low, resulting in reduced bone strength. Moreover, BMD is not uniformly allocated across the different subregions of the proximal femur. These adaptive changes in the morphology of the proximal femur may potentially explain the hip fracture patterns and associated risks. In this direction, dual-energy X-ray absorptiometry (DEXA) has proven to be a valuable tool, making it a routine technique for the quantitative assessment of BMD in vivo. Additionally, BMD is a widely accepted indicator of osteoporosis according to the World Health Organization (WHO) [15,16].

The principal objective of this study is to methodically evaluate whether differences in BMD across various regions of the proximal femur can serve as prognostic indicators for each type of proximal femur fracture. The outcomes derived from this investigation can not only function as a readily applicable and efficient screening tool but also provide a means to refine techniques associated with proximal femoral augmentation. Moreover, these findings may contribute significantly to the enhancement of the diagnostic process for osteoporosis. To accomplish this goal, we employed the widely acknowledged DEXA method. In tandem with this approach, an exhaustive and comprehensive review of the contemporary literature relevant to our thematic focus was also conducted.

## Materials and methods

Inclusion and exclusion criteria

Our study was conducted within the geographical territory of Athens, Greece, and included patients who were admitted to the emergency department and met the criteria outlined in Table 1.

**Table 1:** Inclusion and exclusion criteria of our study. FTF = femoral trochanteric fracture; FNF = femoral neck fracture

Inclusion criteria	Exclusion criteria
Age greater than 60 years	Bilateral hip fractures
Fully ambulatory before the fracture	Hip fractures due to pathological conditions
Diagnosis of either FTF or FNF	Bone tumors or Paget disease
Mechanism of injury was a low-energy side fall	Congenital deformities of the femur and pelvis
	Previous surgical interventions carried out on either the same or contralateral hip, as well as the lower limb in general
	Subtrochanteric fractures

Employing almost identical inclusion and exclusion criteria, we conducted a narrative literature review relevant to our subject. However, we had to exclude studies for which only the abstract was accessible, those published in languages other than English, studies utilizing quantitative CT, and those performed on cadaveric specimens.

Moreover, for the objectives of our study, we made two assumptions. First, despite the primary mechanism of a proximal femoral fracture being a lateral fall impacting the ground [5], we posited the hypothesis that the characteristics of the fall, including direction and force, do not exert any influence on the type of fracture [6]. Second, we assumed that both hips were biomechanically identical.

A total of 70 individuals were included in the study, with 51 females and 19 males. The participants were divided into the following two groups: the FNF (n = 30) and the FTF group (n = 40) based on X-ray assessments. Our research received approval from the Institutional Ethics Committee (reference number: 391). All volunteers provided formal consent to participate in the study, with the agreement that their data would remain anonymous and confidential.

Measurement parameters

**Figure 1:**
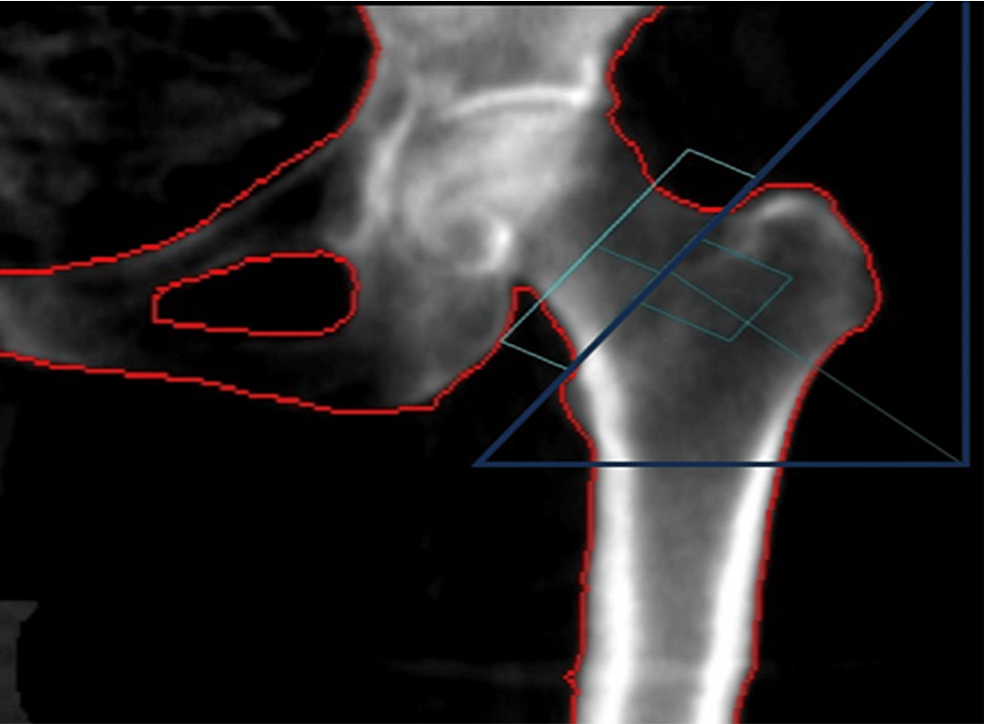
The range of interest of the machine is centered on the femoral neck. A square, measuring 5 cm × 5 cm, was designated as the region of interest for the machine. This square was positioned centrally over the femoral neck area to facilitate the computation of bone mineral density within both its upper and lower halves.

**Figure 2:**
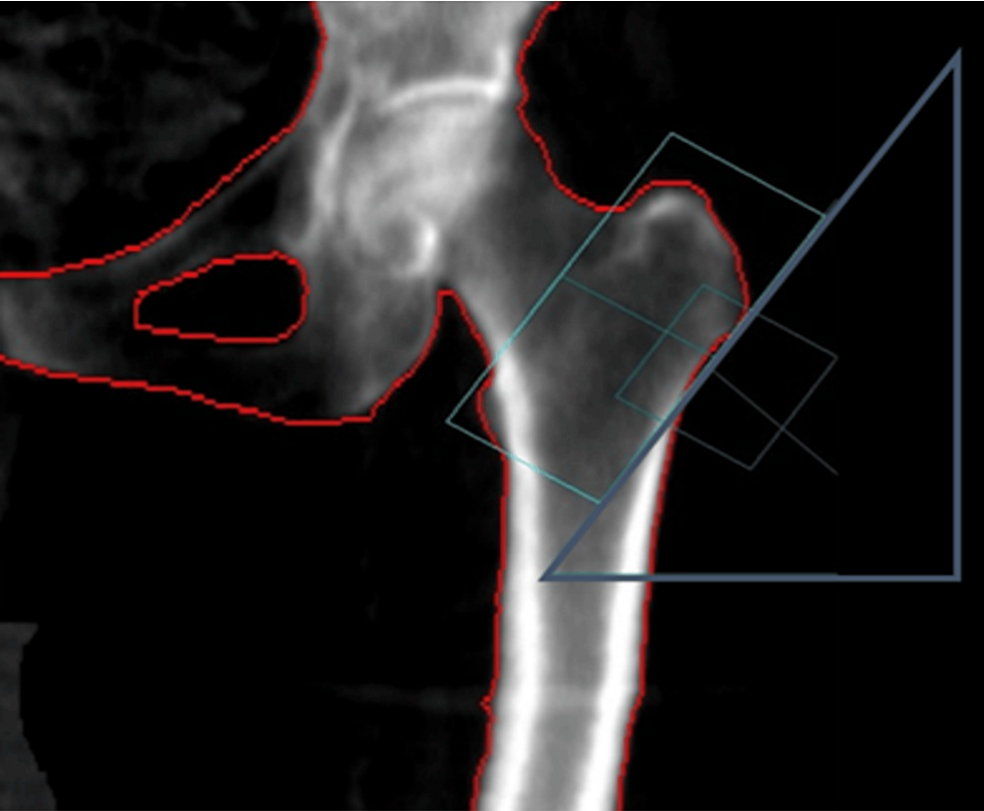
The range of interest of the machine is centered on the trochanteric area. The machine’s designated region of interest was a square measuring 5 cm × 5 cm. Positioned centrally over the trochanteric area, it enabled the calculation of bone mineral density within both its upper and lower halves.

**Figure 3:**
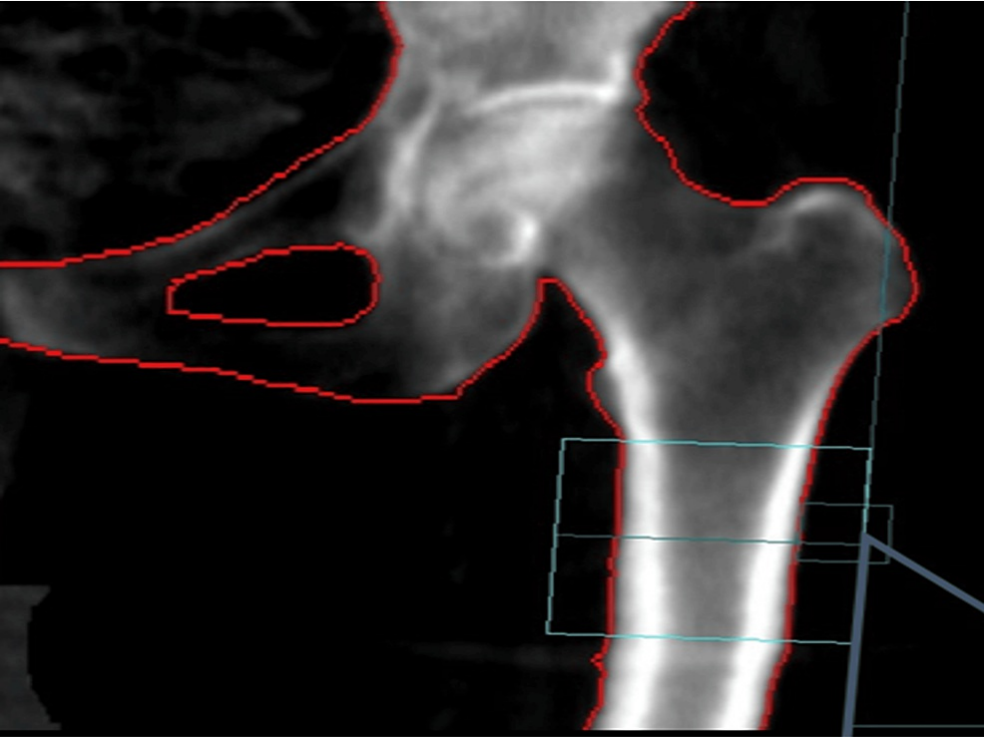
The range of interest of the machine is centered on the area of the femoral shaft, starting from the lowest point of the lesser trochanter. The machine’s designated region of interest was a square measuring 5 cm × 5 cm. Positioned centrally over the femoral neck area and starting from the lower point of the lesser trochanter, it facilitated the calculation of bone mineral density in both its upper and lower halves.

DEXA was employed to measure BMD in various areas of interest within the proximal femur, including the total and upper and lower halves of the femoral neck (Figure 1), the trochanteric region (Figure 2), and the diaphysis (Figure 3).

The region of interest (ROI) of the machine was defined as a square with dimensions 5 cm × 5 cm. Regarding the femoral diaphysis, the measurement commenced from the lowest point of the lesser trochanter. Of note, all measurements were performed once by the same technician using DEXA on the healthy hip of each patient [12]. The Lunar Prodigy apparatus from General Electric Medical Systems was utilized for our research, and the measurements were performed with the patient in a supine position, with the lower limbs positioned in 15 degrees of internal rotation.

Finally, all measurements were conducted within the initial two days after the patient’s admission and before the fixation of their sustained fracture. This meticulous timeframe was chosen with the explicit intention of alleviating the conceivable repercussions stemming from various factors, including, but not limited to, prolonged bed rest, potential surgical delays, insufficient nutritional support, and diminished ambulatory activities. The rationale behind this deliberate choice was to preemptively mitigate any plausible influences these aforementioned variables might have on our study outcomes, particularly concerning the reduction of bone mass.

Statistical analysis

Categorical variables are described as absolute (N) and relative (%) frequencies. Quantitative variables were tested for normality of distribution using the Kolmogorov-Smirnov test. The test indicated that the variables followed a normal distribution. Consequently, data are described as means ± standard deviations. For the comparison of the categorical variables, Pearson’s χ^2^ criterion was used, while Student’s t-test was applied for the comparison of means of quantitative variables across fracture types. The independent-sample Student’s t-test was performed after testing for equality of variances using Levene’s statistic. Pearson’s correlation coefficient was used for bivariate correlations between age and continuous variables. All tests were two-sided. Bonferroni adjustment was used to control type I error inflation due to multiple comparisons, setting the significance level to 0.003. Notably, this adaptation mitigates the occurrence of obtaining a noteworthy outcome solely through random chance. Post hoc power analysis showed that a mean difference in the variable of interest equal to 0.15 with a standard deviation of 0.2 was adequate in our sample of 70 participants, obtaining a statistical power of 0.86. Finally, while linear regression analysis is generally preferred, our specific case posed challenges. Univariate linear regression would yield no discernible divergence from traditional statistical tests in terms of statistical significance. Furthermore, opting for a multiple linear regression model would be statistically inappropriate due to intra-correlation among the explanatory variables assessed in our study. This would introduce considerable complications in interpreting a multiple linear regression model. The statistical package Stata v.17.0 (StataCorp LLC, College Station, TX, USA) was used for data analysis.

## Results

Sample characteristics are shown in Table 2. The majority of our sample (60%) was evaluated as Tönnis grade 2.

**Table 2:** Descriptive statistics of the total sample. The unit of measurement is g/cm^2^.

		Ν	%
Gender	Women	51	72.90%
	Men	19	27.10%
Femoral fracture type	Trochanteric (FTF)	30	42.90%
	Neck (FNF)	40	57.10%
Tönnis classification	0	1	1.43%
	1	6	8.57%
	2	42	60.00%
	3	19	27.14%
	4	3.8	2.86%
		Mean	Standard deviation
Age (year)		80.53	9.04
Total bone mass density (BMD)		0.75	0.15
Total femoral neck BMD		0.68	0.12
BMD of the upper half of the femoral neck		0.50	0.11
BMD of the lower half of the femoral neck		0.85	0.14
Total BMD of the trochanteric region		0.78	0.16
BMD of the upper half of the trochanteric region		0.62	0.15
BMD of the lower half of the trochanteric region		0.92	0.20
Total BMD of the femoral shaft (upper-lower)		1.52	0.35
BMD of the upper half of the femoral shaft		1.42	0.34
BMD of the lower half of the femoral shaft		1.64	0.36
T-score		-2.58	1.08

A comparison of all variables by femoral fracture type is described in Table 3. According to our analytic approach, no measurement differed significantly between the two fracture types. In addition, mean age and both gender and Tönnis classification distribution did not differ significantly when compared by fracture type.

**Table 3:** Analysis by fracture type. The unit of measurement is g/cm^2^. Significant at p-values <0.003.

		Femoral fracture type				
		Trochanteric (FTF)		Neck (FNF)		
		Ν	%	Ν	%	P-value
Gender	Women	24	80.00%	27	67.50%	0.244
	Men	6	20.00%	13	32.50%	
Tönnis classification	0	0	0.00%	1	2.50%	0.271
	1	4	13.33%	2	5.00%	
	2	16	53.33%	26	65.00%	
	3	8	26.67%	11	27.50%	
	4	2	6.67%	0	0.00%	
		Mean	Standard deviation	Mean	Standard deviation	P-value
Age (year)		82.97	8.15	78.70	9.35	0.050
Total bone mass density (BMD)		0.73	0.16	0.77	0.14	0.200
Total femoral neck BMD		0.67	0.13	0.69	0.11	0.598
BMD of the upper half of the femoral neck		0.50	0.12	0.50	0.10	0.813
BMD of the lower half of the femoral neck		0.84	0.15	0.86	0.14	0.485
Total BMD of the trochanteric region		0.75	0.16	0.80	0.16	0.187
BMD of the upper half of the trochanteric region		0.59	0.16	0.64	0.14	0.160
BMD of the lower half of the trochanteric region		0.90	0.19	0.93	0.20	0.464
Total BMD of the femoral shaft (upper-lower)		1.47	0.34	1.57	0.36	0.259
BMD of the upper half of the femoral shaft		1.37	0.33	1.45	0.35	0.324
BMD of the lower half of the femoral shaft		1.57	0.33	1.68	0.38	0.192
T-score		-2.52	1.42	-2.62	0.75	0.704

Analysis by gender is depicted in Table 4. The mean values of several measurement parameters differed significantly between the two genders. More specifically, mean values of total BMD, total femoral neck BMD, total BMD of the trochanteric region, and total BMD of the femoral shaft (upper-lower) were significantly lower in women than in men.

**Table 4:** Analysis by gender. The unit of Measurement is g/cm^2^. *: Significant after Bonferroni correction (p = 0.003).

		Gender				
		Women		Men		
		Ν	%	Ν	%	P-value
Tönnis classification	0	1	1.96%	0	0.00%	0.168
	1	3	5.88%	3	15.79%	
	2	34	66.67%	8	42.11%	
	3	11	21.57%	8	42.11%	
	4	2	3.92%	0	0.00%	
		Mean	Standard deviation	Mean	Standard deviation	P-value
Age (year)		82.59	7.14	75.00	11.30	0.012
Total bone mass density (BMD)		0.72	0.15	0.84	0.11	0.002*
Total femoral neck BMD		0.65	0.12	0.75	0.08	<0.001*
BMD of the upper half of the femoral neck		0.48	0.10	0.57	0.10	0.001*
BMD of the lower half of the femoral neck		0.83	0.16	0.91	0.07	0.007
Total BMD of the trochanteric region		0.74	0.15	0.90	0.13	<0.001*
BMD of the upper half of the trochanteric region		0.57	0.14	0.72	0.12	<0.001*
BMD of the lower half of the trochanteric region		0.87	0.19	1.05	0.17	0.001*
Total BMD of the femoral shaft (upper-lower)		1.42	0.32	1.81	0.29	<0.001*
BMD of the upper half of the femoral shaft		1.33	0.30	1.67	0.31	<0.001*
BMD of the lower half of the femoral shaft		1.52	0.32	1.94	0.29	<0.001*
T-score		-2.76	0.90	-2.09	1.37	0.020

The results of the bivariate correlations of our measurements and age are described in Table 5. Only total BMD of the trochanteric region, BMD of the upper half of the trochanteric region, and BMD of the lower half of the trochanteric region were significantly and inversely correlated with age, although of moderate strength (-0.392, -0.425, -0.407, -0.396; p = 0.001, <0.001, <0.001, and 0.001, respectively). According to the aforementioned associations, as our patients grew older, the specific measurements seemed to decrease.

**Table 5:** Bivariate correlations with age. *: Significant after Bonferroni correction (p = 0.003).

	Pearson’s correlation coefficient	P-value
Hip axis length	-0.392*	0.001
Total bone mass density (BMD)	-0.322	0.007
Total femoral neck BMD	-0.277	0.020
BMD of the upper half of the femoral neck	-0.241	0.044
BMD of the lower half of the femoral neck	-0.258	0.036
Total BMD of the trochanteric region	-0.425*	<0.001
BMD of the upper half of the trochanteric region	-0.407*	<0.001
BMD of the lower half of the trochanteric region	-0.396*	0.001
Total BMD of the femoral shaft (upper-lower)	-0.281	0.018
BMD of the upper half of the femoral shaft	-0.282	0.019
BMD of the lower half of the femoral shaft	-0.288	0.015
Total BMD of the femoral shaft (inner-outer)	-0.289	0.015
BMD of the inner half of the femoral shaft	-0.304	0.010
BMD of the outer half of the femoral shaft	-0.230	0.055
T-score	-0.282	0.018

## Discussion

We employed DEXA to meticulously quantify BMD within specific segments of the proximal femur. In the course of our comprehensive study, adjustments were made to the ROI settings on our apparatus, precisely targeting the femoral neck, trochanteric area, and femoral shaft, with each of these regions further subdivided into upper and lower halves for thorough analysis. The primary hypothesis posited that the intricate mapping of BMD can play a pivotal role in unraveling the underlying etiological mechanisms responsible for femoral neck and trochanteric fractures. However, the statistical analysis of our sample did not produce statistically significant findings. Despite this, the outcomes of our study, the first to encompass a Greek population, carry clinical significance and are aligned with other studies. The results could contribute not only to BMD mapping of the proximal femur and the prediction of hip fractures but also to the ongoing efforts by numerous investigators to augment its more vulnerable areas.

Bone aging is a complex process characterized by a constant balancing of intrinsic bone components. This balance is essential to both resist fractures and optimize skeletal weight. The critical attributes of stiffness and strength, necessary for bearing substantial loads, must be harmonized with toughness or ductility to effectively absorb energy from impact loads. Notably, cortical and trabecular struts undergo weakening with advancing age, contributing to an age-dependent escalation in bone fragility. Moreover, the quantity and distribution of mineral elements and bone cells, including osteocytes, play pivotal roles. Even minor disruptions in these factors can lead to increased micro-damage, which accumulates over time [17,18]. The incapacity to repair these micro-fractures and their subsequent spread results in reduced bone toughness [19]. Wolff’s law further posits that bone shape adapts in response to developmental and aging-related load changes, influencing functional ability [20]. Intriguingly, various studies have reported a 10-fold increase in the 10-year fracture risk for older individuals with almost similar BMD measurements [21]. This observation challenges the traditional assumption that osteoporosis is solely an age-associated disease. Despite the decline in bone mass density and strength with age, not all elderly individuals exhibit skeletal fragility. Furthermore, osteoporosis can affect also young individuals, underlining the complicated synergy of factors influencing bone health beyond the scope of aging.

The trochanteric region and femoral neck region represent two distinct anatomical areas. The first consists approximately of 90% cancellous bone, a proportion that is significantly greater than that of the femoral neck [22]. As a representation of bone strength, BMD decreases gradually throughout the aging process, predominantly affecting the trochanteric region, making it a potential strong predictive factor of FTF [23]. In contrast, from an anatomical perspective, the diameter of the intertrochanteric area is often twice the size of that of the neck [24], creating a moment of inertia that is 16 times lower in the femoral neck when compared to the trochanteric area [25,26]. Consequently, it appears that neck area and subsequently FNF are mainly influenced by geometrical and mechanical factors instead of BMD [27]. Remarkably, a comprehensive understanding of each fracture pattern will contribute to the development of more efficient treatment strategies.

A robust theory posits that as long as the BMD of the trochanteric region remains high, the impact force of a side fall is directed toward the femoral neck, leading potentially to an FNF [28-30]. Conversely, the aging process results in a reduction of BMD within the trochanteric area, causing a decrease in trabecular volume and surface density. Consequently, this depletion in BMD impairs the capacity to absorb and dissipate the energy generated by a lateral fall, ultimately leading to an FTF [31,32]. This hypothesis finds substantive support among various authors [28,30,33-36]. On the contrary, our study, along with the studies conducted by Li et al. [37] and Maeda et al. [38], who employed quantitative computed tomography (qCT), failed to verify this outcome. Furthermore, our research failed to establish a correlation between BMD in the upper and lower halves of the femoral neck and trochanteric regions and the fracture groups. Noteworthy, Johannsdottir et al. [39] and Szulc et al. [25], focusing their studies exclusively on women, mentioned that in cases of FTF, BMD was lower in the femoral neck instead of the trochanteric area.

Quite a few authors have documented a notable observation wherein BMD in the trochanteric region is decreased in cases of severe osteoarthritic hips compared to BMD of the femoral neck region. The same pattern has also been reported for the values of total BMD [40-42]. This finding could be clarified through the application of Wolff’s law and the piezoelectric effect. Specifically, during one- or two-legged stances, the femoral neck experiences compressive loads, whereas the great trochanter primarily sustains tensional loads. In addition, when considering the diminished utilization of the affected hip due to pain and stiffness, it becomes evident that BMD of the trochanteric area suffers an even greater reduction [43-45]. This assumption, when considered alongside the epidemiological data, could elucidate the higher incidence of FTF in cases of high-grade osteoarthritic hips, as classified by Tönnis [46]. In our study involving Greek patients, we were unable to establish a straight correlation between higher Tönnis classification grades and the values of total BMD and trochanteric region BMD and the type of proximal femoral fracture.

Despite our decision to exclude patients with subtrochanteric fractures from our study, we conducted measurements of the BMD in the subtrochanteric region, aiming to investigate a potential correlation with the occurrence of femoral neck or trochanteric fractures. Regrettably, we were unable to establish any such relationship. Furthermore, it is worth mentioning that, to our knowledge, there is an absence of existing literature suggesting an association between the BMD of the subtrochanteric region and proximal femoral fractures.

T-score is defined as the SDs by which a patient’s BMD deviates from the BMD of a reference group consisting of healthy young individuals (30 years old) who are matched for gender and ethnicity and serve as a control group [47]. In their study, Hey et al. [34] aimed to discover a relationship between the total T-score of the hip and BMD in the trochanteric and femoral neck areas with either of the two proximal femoral fracture groups. Their results indicated that although statistically significant differences in regional BMD values were observed between fracture patterns, the mean T-scores measured exceeded the standard osteoporosis threshold, which was defined as -2.5 by the WHO. Therefore, relying only on the T-score might cause an underestimation of the true fracture risk. In our investigation, we did not prove a correlation between Τ-score and proximal femoral fracture type.

In recent years, numerous researchers have been actively investigating the feasibility of prophylactic augmentation in specific regions of the proximal femur to prevent subsequent fractures. The primary skepticism characterizing these studies pertains to the restoration of compromised bone strength. The objective is to enhance the hip’s capacity to absorb and diffuse the impact load caused by a lateral fall, thereby effectively preventing a proximal femoral fracture [48]. The initial step toward achieving this target involves the precise identification of weak and osteoporotic areas. In this context, a predominant approach among researchers involves the utilization of the precise techniques of qCT and finite element modeling. Subsequently, the selection of the most suitable material for augmenting the targeted area is undertaken from a diverse range of options. The commonly explored materials include polymethylmethacrylate bone cement, ceramic-based bone cement, and composite bone grafts. Notably, due primarily to ethical considerations, a majority of studies have been conducted using cadaveric specimens, demonstrating highly promising results [49-53]. An exception to this trend is the work of Howe et al. [52], who performed injections of AGN1, a triphasic, calcium-based, osteoconductive material, into the bone loss area of postmenopausal osteoporotic women. This intervention led to local osteoenhancement, as evidenced by increased BMD and hip strength [52]. In addition, various approaches for the placement of augmented material, either extensive or minimally invasive, have been proposed, always conducted under fluoroscopic imaging [54]. While these methodologies are currently in the early stages of research, requiring additional investigation into their safety, biomechanical efficacy, and resilience, they possess the inherent capability to serve as catalysts for substantial scientific progress in the foreseeable future.

The current diagnostic and therapeutic approaches for osteoporosis heavily rely on the measurement of BMD. The results proposed in this study are expected to augment these decision-making processes. However, it is crucial to underscore that there is currently no official guideline supporting the idea that BMD alone can adequately identify individuals at a heightened risk of fracture [55,56]. Furthermore, since 2008, WHO [57] has introduced the FRAX score, a meticulously designed tool for evaluating the risk of new fractures. This scoring system not only considers BMD and T-score but also incorporates clinical parameters such as gender, age, weight, height, individual or parental history of fracture, rheumatoid arthritis, glucocorticoid intake, alcohol consumption, and smoking status. The aim is to comprehensively consider all potential risk factors and treat the patient as a holistic entity.

Limitations

Several limitations are present in our study. Initially, we assumed that both hips were almost biomechanically identical, implying similar BMD on both sides. Any instance where one side had a history of trauma or bone disease resulted in the exclusion of the patient from the research. Second, it is well-established that BMD tends to be greater in males than in females. Furthermore, our investigation lacked a control group comprising individuals without fractures, which would have facilitated a more robust comparison of our outcomes. Additionally, we did not consider the patients’ medical history and risk factors for osteoporosis. Moreover, our methodology involved the use of the conventional DEXA method rather than qCT. Although the former provides less precision in parameter assessment, it contributes to the simplicity and replicability of our screening tool. The rationale behind this choice was to develop a straightforward and easily reproducible screening tool with broad applicability. Lastly, it is crucial to mention that the size of our sample, consisting primarily of patients of Greek origin, was relatively small due to the strict nature of our inclusion and exclusion criteria. Future studies should seek to address these limitations and underscore the importance of combining both BMD and geometrical characteristics. This approach holds the potential to predict fracture types and associated risks.

## Conclusions

The original objective of this study was to employ the simple and commonly used DEXA method to define whether variations of BMD across specific regions of the proximal femur could serve as a differentiating factor between femoral neck and trochanteric fracture types. The results of this study have the potential to function not only as an effective screening tool, providing insights into the likelihood of future fractures, but also aid in the precise mapping of BMD in the proximal femur. Unluckily, our investigation did not succeed in establishing a direct correlation between our parameters and the type of proximal femoral fracture. This underscores the need for further research endeavors to address this knowledge gap and contribute to the re-establishment of clinical guidelines in the treatment of proximal femoral fractures. In this regard, future studies could consider utilizing qCT as a method. This approach would not only provide a more precise understanding of the internal microarchitecture of the proximal femur but also allow for an investigation into its geometrical configurations. The integration of both intrinsic and extrinsic properties of the proximal femur holds the potential to facilitate timely and early preventive measures against proximal femoral fractures.
